# Grunwald-Winstein Analysis - Isopropyl Chloroformate Solvolysis Revisited

**DOI:** 10.3390/ijms10030862

**Published:** 2009-03-02

**Authors:** Malcolm J. D’Souza, Darneisha N. Reed, Kevin J. Erdman, Jin Burm Kyong, Dennis N. Kevill

**Affiliations:** 1 Department of Chemistry, Wesley College, 120 N. State Street, Dover, Delaware 19901-3875, USA; 2 Department of Chemistry and Applied Chemistry, Hanyang University, Ansan, Kyunggi-do, 425-791, Korea; 3 Department of Chemistry and Biochemistry, Northern Illinois University, DeKalb, Illinois 60115-2862, USA

**Keywords:** Solvolysis, solvolysis-decomposition, nucleophilicity, ionizing power, isopropyl chloroformate, chloroformates, Grunwald-Winstein Equation, Linear Free Energy Relationships

## Abstract

Specific rates of solvolysis at 25 °C for isopropyl chloroformate (**1**) in 24 solvents of widely varying nucleophilicity and ionizing power, plus literature values for studies in water and formic acid, are reported. Previously published solvolytic rate constants at 40.0 °C are supplemented with two additional values in the highly ionizing fluoroalcohols. These rates are now are analyzed using the one and two-term Grunwald-Winstein Equations. In the more ionizing solvents including ten fluoroalcohols negligible sensitivities towards changes in solvent nucleophilicity (*l*) and very low sensitivities towards changes in solvent ionizing power (*m*) values are obtained, evocative to those previously observed for 1-adamantyl and 2-adamantyl chloroformates **2** and **3**. These observations are rationalized in terms of a dominant solvolysis-decomposition with loss of the CO_2_ molecule. In nine of the more nucleophilic pure alchohols and aqueous solutions an association-dissociation mechanism is believed to be operative. Deficiencies in the acid production indicate 2-33% isopropyl chloride formation, with the higher values in less nucleophilic solvents.

## 1. Introduction

**Figure 1.**
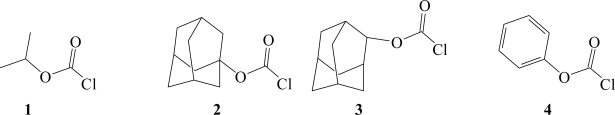
Molecular structures of isopropyl chloroformate (**1**), 1-adamantyl chloroformate (**2**), 2-adamantyl chloroformate (**3**), and phenyl chloroformate (**4**).

Within an ongoing investigation [1] into the utility of the simple [2] and extended (two-term) [3] forms of the Grunwald-Winstein Equation in studies of the mechanism of solvolyses of chloroformate esters, it has previously been shown that the dominant mechanism can vary with the R group of ROCOCl (Figure 1) [1,4–23], with sulfur-for-oxygen substitution [1,10,22,24–28], and with changes in solvent [1,4–29].

In the simple Grunwald-Winstein Equation (Equation 1) [2], *k* and *k_o_* are the specific rates of solvolysis in a given solvent and in the standard solvent (80% ethanol), respectively, *m* represents the sensitivity to changes in the solvent ionizing power *Y* (initially set at unity for *tert*-butyl chloride solvolyses), and *c* is a constant (residual) term. This Equation was developed to correlate the specific

(1) $$\text{log}\;(k/k_{o})=mY+c$$

rates of solvolysis of initially neutral substrates reacting by an ionization (S_N_1 + E1) mechanism. It is now realized both that the scales are leaving-group dependent and that adamantyl derivatives provide better standard substrates, and for a leaving group X a series of *Y_X_* scales are available [30]. For bimolecular (S_N_2 and/or E2) reactions, the correlation is extended (Equation 2) [3] to include a term governed by the sensitivity *l* to changes in solvent nucleophilicity (*N*):

(2) $$\text{log}\;(k/k_{o})=lN+mY+c$$

Initially, Schadt, Bentley, and Schleyer [31] used methyl *p*-toluenesulfonate, to arrive at the *N_OTs_* scale. More recently *N_T_* scales based on the solvolyses of *S*-methyldibenzothiophenium ion [32] have been developed, in which the leaving group is a neutral molecule, which is little influenced by solvent change, and these values [32,33] have become the recognized standards for considerations of solvent nucleophilicity. The magnitudes of the *l* and *m* values can give important indications regarding the mechanism of solvolysis.

**Scheme 1.**
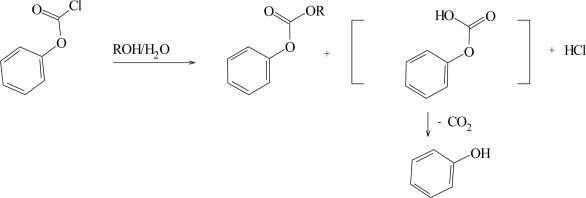
Alcoholysis plus hydrolysis of phenyl chloroformate (**4**).

The solvolyses (Scheme 1) of phenyl chloroformate (**4**) have been extensively studied [7,21] over the full range of the types of solvent usually incorporated into a treatment of the kinetic effect of solvent variation utilizing the extended Grunwald-Winstein Equation (Equation 2). The data are very robust and various sub-sets of values gave essentially the same sensitivity values. A high sensitivity (*l* = 1.66) towards changes in solvent nucleophilicity and a moderate sensitivity (*m* = 0.56) towards changes in solvent ionizing power were observed [21]. These values are now taken as typical values for attack at acyl carbon proceeding by the addition-elimination mechanism, with the addition step rate-determining [1,17,34,35]. The specific rates of solvolysis of methyl chloroformate [12] correlate with similar sensitivity values, except that in the most highly ionizing and weakly nucleophilic of the studied solvents, 90% 1,1,1,3,3,3-hexafluoro-2-propanol (HFIP), a positive deviation was observed, possibly reflecting a superimposed contribution to the postulated association-dissociation (addition-elimination) mechanism from an ionization mechanism.

This superimposed mechanism is observed over a wider range of solvents in the solvolyses of ethyl [10], isopropyl [13], *n*-octyl [16], and *n*-propyl [18] chloroformate esters. These solvolyses could be treated by application of Equation 2 in terms of two correlations with very different sets of *l* and *m* values, believed to correspond to the side-by-side operation of the association-dissociation and ionization pathways.

**Scheme 2.**
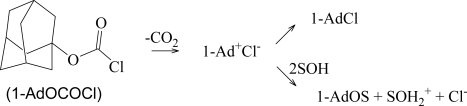
Solvolysis-decomposition of 1-adamantyl chloroformate (**2**).

The simplest chloroformate ester incorporating a tertiary alkyl group, *tert*-butyl chloroformate, decomposes rapidly [36] and cannot be conveniently studied. However, it has been possible to study [4,37] the bridgehead 1-adamantyl chloroformate (**2**) under solvolytic conditions. It was found that in hydroxylic solvents not only the decomposition product but also the solvolysis product involve loss of carbon dioxide, with both processes proceeding at measurable rates (Scheme 2). Application of the simple Grunwald-Winstein Equation (Equation 1), using the original *tert*-butyl *Y* scale, led to a rather low *m* value of 0.62, corresponding to a value of about 0.47 on the *Y*_Cl_ scale (using the approximate interrelationship [30] *Y* = 0.75 *Y*_Cl_). These observations parallel those for 2-adamantyl chloroformate (**3**) [5]. Analyses of the solvolyses of **3** using the extended Grunwald-Winstein Equation (Equation 2) showed that the *l* value (0.03) was negligible and was coupled with an *m* value of 0.48 [5]. This is nicely consistent with a concerted fragmentation-ionization with reduced sensitivities to changes in solvent ionizing power.

**Scheme 3.**
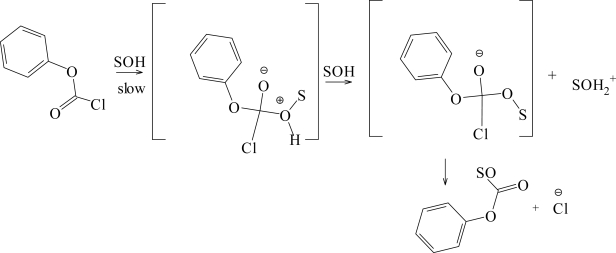
Stepwise addition-elimination mechanism through a tetrahedral intermediate proposed for phenyl chloroformate (**4**).

To summarize, phenyl chloroformate (**4**) reacts by a bimolecular association-dissociation mechanism (Scheme 3). Although simple kinetic studies do not allow one to distinguish, a relatively high kinetic solvent isotope effect in both water and methanol [11] suggests the importance of general-base catalysis and that the first two steps may merge into one or deprotonation of the first formed intermediate is a rate-determining requirement for preventing a rapid reversal to reactants. Methyl chloroformate behaves very similarly, but with a small ionization component in 90% HFIP.

The ionization component is observed over a wider range of solvents for ethyl chloroformate and it becomes almost totally dominant, with accompanying loss of CO_2_, for **2** and **3**. The missing link in these reports of the influence of solvent variation is a detailed study of a chloroformate ester containing a simple secondary alkyl group. Previously published results at 40.0 °C [13] were indicative of a dominant ionization pathway in a majority of solvents studied, except for the least ionizing-more nucleophilic combination of EtOH, 90% EtOH, MeOH, and 90% MeOH. The National Institute for Occupational Safety and Health (NIOSH) has documented on their International Chemical Safety Cards (ICSCs) [38] that isopropyl chloroformate decomposes on heating or on contact with acid, producing toxic and corrosive fumes including chlorine and phosgene. Recently, isopropyl chloroformate like other alkyl haloformate esters are gaining wider importance in the derivatization [39] of highly polar pharmaceuticals in the aqueous phase. Furthermore, in recent studies leading to analyses in terms of Grunwald-Winstein Equations, fluoroalcohols have been shown to be extremely important either as pure solvents or as components of binary mixtures [1,10,16,21–25,34,40–45], in the determination of meaningful *l* and *m* values. Hence in order to bring further clarity, the present investigation reports on the kinetics at 25.0 °C of the solvolyses of isopropyl chloroformate (**1**) including those in solvents having an appreciable fluoroalcohol component, and two additional specific rates in 97% HFIP and 90% HFIP at 40.0 °C, so as to provide a more comprehensive analysis, leading to a better understanding of the solvolytic mechanism for this important missing link.

Other recent studies of aryl [6,8,9,11,17,19,28,35], alkyl [19,28], and alkenyl [20] chloroformate solvolyses have carried out analyses in terms of the bulk concentrations of the components of binary water-alcohol mixtures. A combination of kinetic and product studies strongly suggests for situations involving a rate-determining attack by solvent a process which is kinetically of the third order, involving general-base catalysis by one solvent molecule to the nucleophilic attack of a second molecule. The rather large solvent kinetic isotope effects observed are nicely consistent with the involvement of two solvent molecules in this manner.

### 2. Results and Discussion

The specific rates of acid formation were determined in 24 solvents at 25.0 °C. The solvents consisted of methanol and ethanol, and binary mixtures of water with methanol, ethanol, acetone, 2,2,2-trifluoroethanol (TFE), or HFIP, plus binary mixtures of TFE with ethanol and two values in 97%, 90% HFIP were measured at 40.0 °C. From literature values for the specific rates of solvolysis at several other temperatures, the Arrhenius Equation was used to calculate values at 25.0 °C for solvolyses in water [46] and 99.3% formic acid [47].

The experimental specific rates at 25.0 °C, the two pairs of calculated values, the previously published specific rates at 40.0 °C together with the two additional specific rates in 97% HFIP and 90% HFIP at this temperature, and the *N*_T_ [32,33] and the *Y*_Cl_ values [30,41,43,48,49] are reported in Table 1. In two instances, the experimental rate values were in good agreement with the literature rate values [50,51]. Also contained in Table 1 are percentage values at 40.0 °C for the experimental infinity titer relative to the titer that would have been observed if all reaction had proceeded with acid formation. The deficit indicates the percentage of reaction proceeding with isopropyl chloride formation.

There have been previous studies of the solvolysis of **1** in a few of the solvents listed in Table 1. Leimu [50] studied the methanolysis of a series of chloroformate esters, including the isopropyl ester, and found the specific rates of solvolysis to be in the order: CH_3_OCOCl > CH_3_CH_2_OCOCl ~ CH_3_CH_2_CH_2_OCOCl > **1**. The value reported for the solvolysis of **1** at 25.0 °C is in excellent agreement with the value in Table 1. The mechanism was not considered but the ordering is that to be expected for bimolecular attack by solvent rather than a rate-determining ionization. A similar ordering was also observed for the ethanolyses [51], and an addition-elimination (association-dissociation) mechanism was proposed; the value for ethanolysis of **1** at 25.3 °C is in good agreement (Table 1) with our value at 25.0 °C.

There have also been studies in solvents of considerably higher ionizing power: water [46], 35% acetone [47], and formic acid [47]. Queen studied the hydrolyses of methyl, ethyl, propyl, and isopropyl chloroformates. At a given temperature, the specific rates of hydrolysis in 100% water were found to be in the order: CH_3_OCOCl > CH_3_CH_2_OCOCl ~ CH_3_CH_2_CH_2_OCOCl < **1**. This differs from the ordering observed [50,51] in the alcoholyses in that there is now an increase in going to the isopropyl ester solvolysis. A proposed change from bimolecular to unimolecular mechanism was supported by the observations for the isopropyl chloroformate solvolysis of a considerably more positive entropy of activation and a lower solvent isotope effect than the very similar values observed for the methyl and ethyl esters.

The ordering observed in water was also observed [47] in 35% acetone (65% water) and in 99% formic acid, with a seven-fold faster reaction for **1** than for ethyl chloroformate in the aqueous acetone mixture, increasing to a 70-fold faster reaction in formic acid. It was suggested that both of the esters react by the ionization pathway in formic acid. In support, a recent estimate [12] suggests that the formolysis of ethyl chloroformate proceeds by about 10% addition-elimination and 90% ionization.

The correlations carried out in earlier studies, comparing methyl, ethyl, and isopropyl chloroformate solvolyses, have strongly indicated that the mechanism for **1** is solvent dependent, with a bimolecular association-dissociation pathway favored in pure alcohols, and an ionization pathway favored in the water-rich solvents and in formic acid. In the present investigation we carry out a systemic study of the effect of solvent variation, with the inclusion of solvents rich in fluoroalcohol (TFE and HFIP), allowing a very thorough investigation of the region within which the ionization pathway is expected to operate. Recently, there has been an interesting study considering the relationship between solvolysis rates in 97% TFE for carboxylic and sulfonic acid chlorides and the heterolytic bond dissociation enthalpies for transfer of chloride ion to the acetyl cation [28].

For a number of haloformate esters [1,16,52–55], a consideration of the F:Cl rate ratio has provided some of the best evidence in favor of the addition step within an association–dissociation (addition– elimination) mechanism being rate determining. Since the C–F bond is much stronger than the C–Cl bond, ratios of well below unity would have been anticipated if the carbon–halogen bond was appreciably broken at the transition state of the rate determining step. In contrast, if the addition-step is rate-determining, values close to unity (and frequently above it), reflecting a large electron deficiency at the carbonyl carbon of a haloformate [16] incorporating chlorine are frequently observed. The *k*_F_/*k*_Cl_ ratios reported [54] for isopropyl fluoroformate and chloroformate at 40.0 °C in non-fluoralcohol containing solvents range from a low of 0.2 in pure ethanol to 2.4 in 70% methanol. For these two substrates the common aqueous fluoroalchohol (70% TFE) has a *k*_F_/*k*_Cl_ value of 0.067. The low ratio in 70% TFE follows from the solvolysis of the fluoroformate being by the addition-elimination pathway [54] but this pathway constituting only a minor component in the corresponding solvolysis of the chloroformate [13], which is dominated by the superimposed ionization pathway.

In Table 2 details are given as to how the experimental specific rates of reaction for the solvolysis of **1** at 40.0 °C and 25.0 °C, are corrected for contributions from the ionization pathway leading to an estimate of the contribution from the addition-elimination pathway. The specific rates reported in Table 1, have been analyzed using Equations 1 and 2, and these results are reported in Table 3. Previously reported analyses of methyl chloroformate [12], ethyl chloroformate [10], **4** [7,21], **3** [5], and phenyl chlorodithioformate [21,24], are reported in Table 4, together with new analyses of the data previously presented [4,37] for **2**

**Figure 2.**
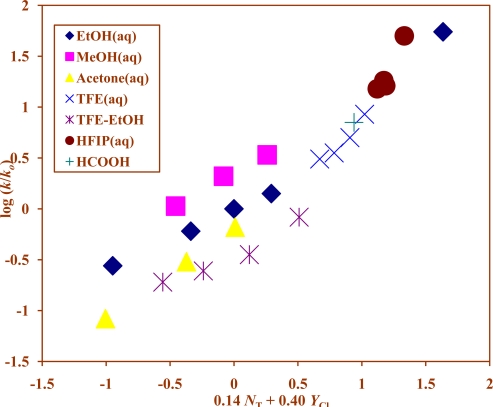
The plot of log (*k/k_o_*) vs. (0.14 *N_T_* + 0.40 *Y_Cl_*) for the solvolyses of isopropyl chloroformate (**1)** in pure and binary solvents at 25.0 ºC.

Use of Equation 1 with all 26 available data points at 40.0 °C leads to a fair correlation with an *m* value of 0.33, and a value of 0.31 is obtained for the 24 data points at 25.0 °C. Using Equation 2 for the data at 40.0 °C, values are obtained of 0.21 for *l* and 0.44 for *m*, and the *F*-test value falls modestly from 130 to 100. Similarly analysis of data at 25.0 °C yields a value of 0.14 for *l*, 0.40 for *m* (Figure 2), and the *F*-test fell from 127 to 71. Since earlier work [46,47] had suggested a duality of mechanism for the solvolyses of isopropyl chloroformate, the fairly good one-term analysis and the low value for *l* in the two-term analysis suggests that, for the majority of the solvent systems, the ionization mechanism is dominant.

Accordingly, it was felt that it would be safe to assess the correlation parameters for the ionization mechanism using specific rates in the seven most highly ionizing solvents: three TFE-H_2_O mixtures, two HFIP-H_2_O mixtures, H_2_O, and HCOOH. It can be seen from the two entries in Table 3 that the two-term Equation gives the best correlation, with values (for data at 40.0 °C), of 0.21 for *l* and 0.58 for *m*; and 0.12 for *l* and 0.60 for *m* (for data at 25.0 °C). The *l* and *m* values barely changes on inclusion of the four TFE-EtOH mixtures and are in reasonable agreement with others believed to be for ionization reaction in solvolyses of chloroformate esters. The low *l* value suggests that solvation involved is at the incipient alkyl carbocation rather than at the acyl carbon. This view is, however, inconsistent with the conclusions reached by Green and Hudson [56] that the solvolysis of secondary alkyl chloroformates proceed via a carboxylium ion (ROCO^+^) intermediate. They found that the rates of formolysis and hydrolysis in aqueous acetone of a series of cycloalkyl chloroformates were slightly larger than the corresponding solvolysis of **1** and increased steadily as one went from five- to eight-membered rings. This is a different ordering to the solvolysis of the cycloalkyl tosylates, where the rate order C_5_>C_6_<C_7_<C_8_ was established and ascribed to internal strain effects [57]. This difference led to the postulate [56] that a rate-determining formation of ROCO^+^, rather than R^+^, was involved in the chloroformate ester solvolysis. It is possible, even if the ionization is indeed to R^+^, that the I-strain effects are not dominant when the transition state is earlier than for the acetolyses of the tosylates, but clearly additional studies are needed in order to give a more detailed picture of the ionization process.

At 40.0 °C, for the 10 solvents that can strongly hydrogen bond (nine fluoroalcohols and formic acid), values are obtained of 0.05 for *l* (0.67 probability that the *lN*_T_ term is statistically insignificant) and 0.41 for *m*, with a correlation coefficient of 0.984 and *F*-test value of 105. The *F*-test value improves significantly to 232 using the one-term Equation, and a low *m* value of 0.37 is obtained. A similar trend is observed for the data set at 25.0 °C. In this instance for the 12 fluoroalcohols and formic acid, analyses using Equation 2 results in a value of 0.05 for *l* (0.66 probability that the *lN*_T_ term is statistically insignificant), a value of 0.43 for *m*, and a *F*-test value of 82. The *F*-test value improves significantly to 175 with Equation 1, and a value of 0.40 is obtained for *m*.

The low *m* values observed at the two experimental temperatures of **1** are reminiscent of the behavior of **2** and **3**, where the solvolysis-decomposition correlates well with the Equation 1, with an *m* value of 0.57 for **2**, and 0.47 for **3** (Table 4). A low *m* value of 0.46 (using *Y*_OTs_ values [30]) was also reported for the solvolyses of 2-adamantyl azoxytosylate, which similarly gives solvolysis and decomposition products, after loss of N_2_O [58]. It was suggested [4, 5, 37, 58] that these low *m* values are a feature of the loss of the small molecule (CO_2_ or N_2_O) during the ionization-carbocation capture process. For **2** there is a 0.71 probability and for **3**, a 0.70 probability, that the *lN*_T_ term is statistically insignificant.

**Figure 3.**
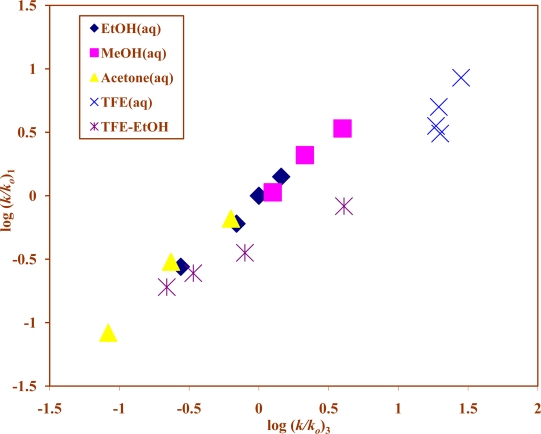
The plot of log (*k/k_o_*) for isopropyl chloroformate (**1**) against log (*k/k_o_*) for 2-adamantyl chloroformate (**3**) in pure and binary solvents at 25.0 ºC.

The similarity in *l* and *m* values for **1** and **3** suggest that a very good direct linear relationship exists between their specific rates of solvolysis, which is further substantiated by an inspection of the plot shown in Figure 3 of log (*k*/*k_o_*) values for **1** against those for **3**, with a correlation coefficient of 0.945, *F-*test value of 132, slope of 0.68 ± 0.06, and an intercept of −0.16 ± 0.05.

The low *m* value for the ionization pathway, similar to that for the bimolecular pathway, means that the division of the solvolyses into the two mechanistic categories is not as clear-cut as it was for EtOCOCl [10], PhSCOCl [21,25], and PhSCSCl [21,24]. The solvolysis of **4** (*l* = 1.66; *m* = 0.56) and PhSCSCl (*l* = 0.69; *m* = 0.95) can be taken as models for mechanisms involving addition-elimination and ionization without fragmentation, respectively. In Table 2 we report the experimental specific rates of reaction as corrected for the calculated specific rates for the ionization pathway, obtained using the *l*, *m*, and *c* values from Table 3 and the *N*_T_ and *Y*_Cl_ values from Table 1. These (*k* − *k*_i_) values are then used to calculate the correlation parameters for the addition-elimination pathway in the nine solvents for which it dominates (Table 3). The *l* value of 1.35 ± 0.22 at 40.0 °C, and 1.01 ± 0.26 at 25.0 °C are approximately similar to the value of 1.66 ± 0.05 for **3** and the addition-elimination pathway values of 1.59 ± 0.0.09 for MeOCOCl solvolysis and 1.56 ± 0.09 for EtOCOCl solvolysis. The higher standard error results from the much narrower range of operation of the mechanism for the solvolysis of **1**. Indeed, the similarity in character of the nine solvents involved would be expected to lead to an appreciable degree of multicollinearity when Equation 2 is applied to the data. Minor changes in specific rate values can then lead to relatively large changes in sensitivity values.

Only one solvent (20T-80E) has such a well-balanced component from each mechanism that it does not appear in each correlation. The remaining 16 solvents at 40.0 °C give a correlation (Table 3) with parameters very similar to those for the seven highest ionizing power solvents (*l* = 0.28; *m* = 0.59), consistent with the previously expressed belief that the ionization pathway is dominant in the majority of the studied solvents. Both the *l* and *m* values are lower than for the solvolyses of PhSCSCl, suggesting an earlier transition state.

An alternative, and more gentle way of investigating the kinetic effect of solvent variation is by studying the kinetic solvent isotope effect (KSIE), usually involving replacement of hydrogen by deuterium. In his study of the hydrolysis of several chloroformate esters, Queen [46] studied the KSIE when H_2_O was replaced by D_2_O. The effect is usually considered in terms of the *k*_H_2_O_/*k*_D_2_O_ ratio. For methyl, ethyl, and phenyl esters, he obtained very similar specific rate ratios of 1.89 (at 25.0 °C), 1.82 (at 25.0 °C), and 1.79 (at 7.5 °C). For **1**, the specific rate ration fell to 1.25 (at 25.0 °C). This was considered to reflect a change from a bimolecular process to a unimolecular process. This finding is nicely consistent with the present study, where an analysis indicates that, for the hydrolysis in 100% water, essentially all of the reaction proceeds by the ionization mechanism (Table 2).

The kinetic runs with addition of the neat liquid substrate were carried out using carefully measured amounts of substrate and it is possible to compare the experimental infinity titers with the theoretical values. A comparison by Queen for the reaction in water [46] has previously given a value of 98%, which would correspond to 2% isopropyl chloride formation. The values obtained are reported in Table 1. In 100%–60% methanol and 100%–50% ethanol, we also observe fairly close to the theoretical value for 100% solvolysis, with values in the range of 87 ± 5% to 97 ± 1%. Values were slightly lower in 90%–60% acetone, with an acid production range of 78 ± 3% to 86 ± 5%. Probably, a large, relatively inert acetone component to the solvent increases the probability that the isopropyl carbocation will react with the counterion, rather than with a solvent molecule. Similarly, in solvents rich in TFE (97%–70%), the solvent is of reduced nucleophilicity and lower values are observed, with the lowest value for all the solvents studied being 67 ± 1% in 97% TFE. It is probable that even lower values would have been obtained in the HFIP-H_2_O mixtures, but, because of the relatively fast reaction, we concentrated on rapid mixing rather than accurate measurement of the amount of added substrate. Even with accurate measurement, an appreciable amount of product formation would occur, in these solvents, under the inhomogeneous conditions present during the mixing process.

## 3. Conclusions

The Grunwald-Winstein Equations are versatile tools that can be effectively used to gauge solvent effects in solvolysis reactions. The presently reported analyses demonstrate that meaningful contributions associated with the Grunwald-Winstein treatment of isopropyl chloroformate (**1**) can result when an adequate selection of solvents with considerably different *N_T_* and *Y_Cl_* values are made available. Although the analyses are only approximate, due to only a low number of similar solvents having an appreciable contribution from the pathway, the *l* and *m* parameters obtained for **1** in nine of the more nucleophilic solvents are very similar to other chloroformate esters such as phenyl chloroformate (**4**), where the addition step of an addition-elimination pathway is rate-determining. Surprisingly, **1 2**, and **3**, behave very similarly in the solvents that can strongly hydrogen bond, resulting in negligible values of *l* and very low *m* values. This is considered to be indicative of a unimolecular fragmentation-ionization pathway, in which ionization is accompanied by loss of carbon dioxide.

## 4. Experimental

The isopropyl chloroformate (Sigma-Aldrich, 1.0 M in toluene) was used as received. Solvents were purified and the kinetic runs carried out as described previously [42]. A substrate concentration of approximately 0.005 M in a variety of solvents was employed. For some of the runs, calculation of the specific rates of solvolysis (first-order rate coefficients) was carried out by a process in which the conventional Guggenheim treatment [59] was modified [60] so as to give an estimate of the infinity titer, which was then used to calculate for each run a series of integrated rate coefficients. The specific rates and associated standard deviations, as presented in Table 1, are obtained by averaging all of the values from, at least, duplicate runs. Multiple regression analyses were carried out using the Excel 2007 package from the Microsoft Corporation, and the SigmaPlot 9.0 software version from Systat Software, Inc., San Jose, CA, was used for the Guggenheim treatments.

## Figures and Tables

**Table 1.** Specific rates of solvolysis (*k*)^a^ of **1**, in several binary solvents at 25.0 ºC and 40.0 °C; literature values for (*N_T_*) and (*Y_Cl_*); and the percentages of overall reaction proceeding with acid formation at 40.0 °C.

**Solvent (%)*b***	**1 @ 25.0 °C; 10^5^*k*, s^−1^***c,d*	**1 @ 40.0 °C; 10^5^*k*, s^−1^***e*	***N_T_^e^***	***Y_Cl_^f^***	**% Acid***c,f*
100% MeOH	4.19 ± 0.10*g*	15.4 ± 0.1	0.17	−1.2	96 ± 2
90% MeOH	8.28 ± 0.09	30.7 ± 0.6	−0.01	−0.20	97 ± 1
80% MeOH	13.4 ± 0.4	49.7 ± 0.6	−0.06	0.67	88 ± 3
70% MeOH		76.6 ± 0.8	−0.40	1.46	94 ± 2
60% MeOH		120 ± 6	−0.54	2.07	91 ± 2
100% EtOH	1.09 ± 0.04*h*	5.41 ± 0.01	0.37	−2.50	93 ± 3
90% EtOH	2.36 ± 0.09	10.8 ± 0.1	0.16	−0.90	91 ± 2
80% EtOH	3.92 ± 0.14	18.6 ± 0.2	0.00	0.00	91 ± 1
70% EtOH	5.53 ± 0.26	31.3 ± 0.1	−0.20	0.80	89 ± 2
60% EtOH		51.3 ± 0.1	−0.39	1.38	87 ± 5
50% EtOH		96.5 ± 0.3	−0.58	2.02	90 ± 2
90% Acetone	0.331 ± 0.025	1.08 ± 0.03	−0.35	−2.39	78 ± 3
80% Acetone	1.19 ± 0.04	4.27 ± 0.03	−0.37	−0.80	86 ± 5
70% Acetone	2.59 ± 0.08	11.8 ± 0.2	−0.42	0.17	81 ± 2
60% Acetone		28.8 ± 0.2	−0.52	1.00	85 ± 1
97% TFE (w/w)	12.3 ± 0.3	71.6 ± 0.7	−3.30	2.83	67 ± 1
90% TFE (w/w)	13.9 ± 0.4	74.7 ± 0.8	−2.55	2.85	72 ± 1
70% TFE (w/w)	19.7 ± 0.7	117 ± 3	−1.98	2.96	80 ± 3
50% TFE (w/w)	33.5 ± 0.04		−1.73	3.16	
80T-20E	3.26 ± 0.07	21.3 ± 0.7	−1.76	1.89	68 ± 1
60T-40E	1.41 ± 0.10	7.87 ± 0.06	−0.94	0.63	72 ± 1
40T-60E	0.964 ± 0.023	3.76 ± 0.03	−0.34	−0.48	
20T-80E	0.757 ± 0.014	3.16 ± 0.06	0.08	−1.42	
100% H_2_O	218 ± 1*i*	1622 ± 4*i*	−1.38	4.57	98*j*
99.3% HCOOH	28.2 ± 0.1*k*	172 ± 2*k*	−2.44*l*	3.20*l*	
97%HFIP (w/w)	146 ± 2	563 ± 16*d,m*	−5.26	5.17	
90%HFIP (w/w)	63.2 ± 2.9	246 ± 12*d,m*	−3.84	4.41	
70%HFIP (w/w)	60.1 ± 2.4		−2.94	3.83	
50%HFIP (w/w)	71.0 ± 3.0		−2.49	3.8	

^a^ Sum of solvolysis, propene formation, and isopropyl chloride formation.

^b^ Substrate concentration of *ca.* 0.0052 M; binary solvents on a volume-volume basis at 25.0 °C, except for TFE-H_2_O and HFIP-H_2_O solvents which are on a weight-weight basis. T-E are TFE-ethanol mixtures.

^c^ With associated standard deviation.

^d^ Isopropyl chloroformate was added as a 1 M solution in toluene.

^e^ Ref. 13.

^f^ Ratio (as percentage) of the experimental infinity titer and the theoretical (complete acid formation) infinity titer at 40.0 °C.

^g^ A value of 4.10 X 10^−5^ s^−1^ has been reported [50].

^h^ A value 1.04 (± 0.08) X 10^−5^ s^−1^ at 25.3 °C has been reported [51].

^I^ Calculated from 11 values in the 0.5–24.5 °C range [46].

^j^ At 25.0 °C [46].

^k^ Calculated from values at 25.3 °C, 33.0 °C, and 50.6 °C [47].

^l^ Value for 100% HCOOH.

^m^ This study.

**Table 2.** Subtraction of the calculated specific rate by the ionization mechanism (*k*_i_) from the experimentally measured specific rates of reaction (*k*) at 40.0 °C and 25.0 °C.

**Solvent (%)*a***	**10^5^*k*, s^−1^*b***	**10^5^*k*_i_, s^−1^*c***	**10^5^ (*k–k*_i_), s^−1^**	**10^5^*k*, s^−1^*e***	**10^5^*k*_i_, s^−1^*f***	**10^5^ (*k–k*_i_), s^−1^**
100% MeOH	15.4	1.5	13.9*d*	4.19	0.12	4.07*g*
90% MeOH	30.7	5.1	25.6*d*	8.28	0.46	7.82*g*
80% MeOH	49.7	16.2	33.5*d*	13.4	1.53	11.9*g*
70% MeOH	76.6	39.5	37.1			
60% MeOH	120	83.2	36.8			
100% EtOH	5.41	0.29	5.1*d*	1.09	0.021	1.07*g*
90% EtOH	10.8	2.2	8.6*d*	2.36	0.18	2.18*g*
80% EtOH	18.6	6.8	11.9*d*	3.92	0.61	3.31*g*
70% EtOH	31.3	17.8	13.5	5.53	1.73	3.8
60% EtOH	51.3	35.5	15.8			
50% EtOH	96.5	76.1	20.4			
90% Acetone	1.08	0.30	0.78*d*	0.33	0.020	0.31*g*
80% Acetone	4.27	1.93	2.34*d*	1.19	0.18	1.01*g*
70% Acetone	11.8	6.9	4.9*d*	2.59	0.680	1.91*g*
60% Acetone	28.8	19.9	8.9			
97% TFE (w/w)	71.6	60.4		12.3	12.2	
90% TFE (w/w)	74.7	87.6		13.9	15.4	
70% TFE (w/w)	117	134		19.7	21.1	
50% TFE (w/w)				33.5	29.6	
80T-20E	21.3	36.0		3.26	5.08	
60T-40E	7.87	9.9		1.41	1.12	
40T-60E	3.76	3.03	0.7	0.96	0.280	0.68
20T-80E	3.16	1.07	2.1	0.757	0.088	0.67
100% H_2_O	1622	1514		218	229	
99.3% HCOOH	172	150		28.2	25.7	
97%HFIP (w/w)	563	523		146	179	
90%HFIP (w/w)	246	331		63.2	80.0	
70%HFIP (w/w)				60.1	54.1	
50%HFIP (w/w)				71.0	58.1	

^a^ See footnote *b* in Table 1.

^b^ From Table 1, at 40.0 °C.

^c^ Calculated using the Equation log (*k*/*k*_o_) = 0.21 *N*_T_ + 0.58 *Y*_Cl_ – 0.44.

^d^ Values used in the analysis at 40.0 °C of the bimolecular pathway with adjusted specific rate values.

^e^ From Table 1, at 25.0 °C.

^f^ Calculated using the Equation log (*k*/*k*_o_) = 0.12 *N*_T_ + 0.60 *Y*_Cl_ – 0.81.

^g^ Values used in the analysis at 25.0 °C of the bimolecular pathway with adjusted specific rate values.

**Table 3.** Correlation of the specific rates of reaction of **1**, at 40.0 °C and 25.0 °C, using the simple or extended Grunwald-Winstein Equations (Equations 1 and 2).

**Substrate**	***na***	***lb***	***m^b^***	***c^c^***	***R^d^***	***F^e^***
**1;** 40.0 °C	26*f*		0.33 ± 0.03	−0.11 ± 0.07	0.919	130
		0.21 ± 0.06	0.44 ± 0.04	−0.02 ± 0.06	0.947	100
	7*g*		0.44 ± 0.12	−0.56 ± 0.44	0.861	14
		0.21 ± 0.04	0.58 ± 0.05	−0.44 ± 0.16	0.987	74
	11*h*		0.41 ± 0.04	−0.45 ± 0.12	0.964	117
		0.20 ± 0.06 (0.014)*i*	0.53 ± 0.05	−0.33 ± 0.09	0.984	121
	10*j*		0.37 ± 0.02	−0.43 ± 0.07	0.983	232
		0.05 ± 0.11 (0.67)*i*	0.41 ± 0.08	−0.41 ± 0.10	0.984	105
	9*k*		0.36 ± 0.10	0.04 ± 0.14	0.803	13
		1.05 ± 0.17	0.44 ± 0.04	0.15 ± 0.06	0.976	61
	9*k,l*		0.31 ± 0.13	0.03 ± 0.17	0.661	5
		1.35 ± 0.22	0.40 ± 0.05	0.18 ± 0.07	0.960	35
	16*m*		0.38 ± 0.05	−0.28 ± 0.13	0.911	69
		0.28 ± 0.04	0.59 ± 0.04	−0.32 ± 0.06	0.982	176
	
**1;** 25.0 °C	24*f*		0.31 ± 0.03	−0.10 ± 0.07	0.923	127
		0.14 ± 0.08 (0.10)*i*	0.40 ± 0.05	−0.02 ± 0.08	0.933	71
	10*g*		0.52 ± 0.06	−0.83 ± 0.22	0.952	78
		0.12 ± 0.02	0.60 ± 0.03	−0.81 ± 0.10	0.991	203
	14*h*		0.41 ± 0.03	−0.48 ± 0.10	0.967	175
		0.11 ± 0.07 (0.14)*i*	0.48 ± 0.05	−0.41 ± 0.10	0.973	99
	13*j*		0.40 ± 0.03	−0.46 ± 0.09	0.970	176
		0.05 ± 0.10 (0.66)*i*	0.43 ± 0.08	−0.44 ± 0.11	0.971	82
	9*k*		0.36 ± 0.10	0.10 ± 0.13	0.812	14
		0.92 ± 0.23	0.42 ± 0.06	0.20 ± 0.08	0.953	30
	9*k,l*		0.34 ± 0.11	0.11 ± 0.14	0.765	10
		1.01 ± 0.26	0.41 ± 0.06	0.22 ± 0.08	0.940	23

^a^ Using data at 40.0 °C from Table 1 and Table 2; *n* is the number of solvents.

^b^ With associated standard error.

^c^ Accompanied by standard error of the estimate.

^d^ Correlation coefficient.

^e^ *F*-test value.

^f^ All solvents.

^g^ TFE-H_2_O, HFIP-H_2_O, HCOOH, H_2_O

^h^ TFE-H_2_O, TFE-EtOH, HFIP-H_2_O, HCOOH, H_2_O.

^i^ Probability that the contribution to the linear free energy relationship is insignificant.

^j^ TFE-H_2_O, TFE-EtOH, HFIP-H_2_O, HCOOH.

^k^ 100%, 90%, 80% MeOH and EtOH, and 90%, 80%, 70% acetone.

^l^ Specific rate values adjusted as reported in Table 2.

^m^ Remaining solvents after elimination of those listed in footnote *k*, plus omission of 20T-80E.

**Table 4.** Correlation of the specific rates of reaction of other chloroformate esters using the simple or extended Grunwald-Winstein Equations (Equations 1 and 2).

**Substrate**	***na***	***lb***	***mb***	***cc***	***Rd***	***Fe***
EtOCOCl*f*	28	1.56 ± 0.09	0.55 ± 0.03	0.19 ± 0.24	0.967	179
	7	0.69 ± 0.13	0.82 ± 0.16	−2.40 ± 0.27	0.946	17
MeOCOCl*g*	19	1.59 ± 0.09	0.58 ± 0.05	0.16 ± 0.07	0.977	
**4***h*	49	1.66 ± 0.05	0.56 ± 0.03	0.15 ± 0.07	0.980	568
PhSCSCl*i*	31	0.69 ± 0.05	0.95 ± 0.03	0.18 ± 0.05	0.987	521
**2***j*	11		0.57 ± 0.03	0.05 ± 0.08	0.985	294
	11	0.08 ± 0.20 (0.71)*i*	0.59 ± 0.05	0.06 ± 0.08	0.985	133
**3***k*	19		0.47 ± 0.03	−0.11 ± 0.19	0.970	274
	19	0.03 ± 0.07 (0.70)*i*	0.48 ± 0.04	−0.10 ± 0.19	0.971	130

^a^ *n* is the number of solvents.

^b^ With associated standard error.

^c^ Accompained by standard error of the estimate.

^d^ Correlation coefficient.

^e^ *F*-test value.

^f^ Values taken from [10].

^g^ Values taken from [12].

^h^ Values taken from [21].

^i^ Values taken from [21].

^j^ Calculated from the specific rates of reaction in 100%, 95%, 90% methanol; 100%, 95%, 90%, 80% ethanol; 10T-90E, 20T-80E, 30T-70E, and 40T-60E at 25.0 °C [4, 37]. Interpolated values for *N*_T_ and *Y*_Cl_: 0.10 and −0.67 for 95% MeOH; 0.27 and −1.61 for 95% EtOH; −0.33 and −1.51 for 85% acetone; and 0.22 and −1.97 for 10%TFE-90%EtOH. Also an interpolated *Y*_Cl_ value of −0.93 for 30T-70E.

^k^ Values taken from [5].
